# A review of Leila Rose Foundation support for families affected by rare childhood cancer in Australia over the past decade

**DOI:** 10.1002/cnr2.1381

**Published:** 2021-05-03

**Authors:** Gemma Sutherland, Andrew Chow, Tracy Chow, Christopher Broadley

**Affiliations:** ^1^ Family Support Coordinator, Leila Rose Foundation and Employed as Cancer Care Navigator at Warringal Private Hospital Heidelberg Victoria Australia; ^2^ Joint Founding Director Leila Rose Foundation and Practice Partner at the Cambourne Medical Clinic Warrnambool Victoria Australia; ^3^ Joint Founding Director, Leila Rose Foundation Warrnambool Victoria Australia; ^4^ CEO, Leila Rose Foundation and Business Unit Manager, ANZ at ITL Australia Pty Ltd, an Australian Medical Device Manufacturer Melbourne Victoria Australia

## Abstract

**Background:**

The Leila Rose Foundation (“the Foundation”) was established in April 2011, to address financial toxicity as well as the gaps in knowledge and support for families affected by a rare childhood cancer diagnosis in Australia.

**Aim:**

The aim of this brief report is to analyze the diagnostic trends surrounding the rare cancer diagnoses for patients referred to the Foundation over the past decade and to present case studies evaluating the role of the Foundation's Family Support Coordinator in providing tailored, individualized support for families.

**Methods:**

Eligibility for family support is restricted to children ≤ 14 years of age at diagnosis with a cancer that has an incidence less than 5% of all childhood cancers in Australia as reflected by national registry data. The analysis of diagnostic trends in this report, was based upon a systematic review of enrolment records. The role of the Family Support Coordinator is presented in four different case studies.

**Results:**

As at 1 November 2020, the Foundation has supported 197 families affected by rare childhood cancer. Financial support of $825,000 has been provided directly to these families. Enrollment records demonstrate that 35 patients representing 18% of all enrollments have had a unique diagnosis that has not been recorded for any other enrolled patient highlighting that these diagnoses are very rare. The most frequent diagnoses have included Medulloblastoma, Ewing's Sarcoma and Wilm's Tumor (20, 19, 19 patients respectively). The Family Support Coordinator role has provided individualized support for families which has been greatly appreciated based upon ad hoc family feedback.

**Conclusions:**

Challenges remain in terms of improving outcomes for families affected by rare childhood cancer. The Foundation is committed to leaving no stone unturned and delivering its unique support services to families in order to reduce the burden caused by a rare childhood cancer diagnosis both now and in the future.

## 1 BACKGROUND

The Leila Rose Foundation (“the Foundation”) was established in 2011 by Dr Andrew and Tracy Chow following the passing of their daughter Leila Rose, from a rare childhood cancer. Based in Warrnambool, in the state of Victoria, Australia, the Foundation was set up specifically to provide support for families affected by rare childhood cancer. Rare childhood cancer is defined by the Foundation as a cancer with an incidence less than 5% of all childhood cancers in Australia, in children ≤14 years of age at diagnosis.^1^, ^2^ Due to the low numbers and great heterogeneity, rare childhood cancers are difficult to study in a systematic way and tend to lag in terms of funding and resource support. The philosophy of the Foundation is to leave no stone unturned in supporting families affected by rare childhood cancer.

This article reviews the impact of the Foundation over 10 years, provides some themes emerging from the data associated with the children for whom the Foundation has provided support and presents cases, which illustrate the diversity of support provided. The article also highlights some challenges and opportunities for the management of rare childhood cancer in the future.

## 2 INTRODUCTION

Leila Rose (pronounced Layla) was 10 months old when she was diagnosed with a rare and aggressive rhabdoid tumor in 2009. After her initial treatment regimen failed, a subsequent experimental regimen showed real promise as her tumor reduced in size and general health improved. Tragically the tumor reduction was temporary and the cancer regrew resulting in Leila losing her battle at the age of only twenty‐one months.

After the passing of Leila, her parents wondered how many other families had experienced this turmoil and how many are still suffering through it, and with this they established the Leila Rose Foundation in 2011, in memory of their daughter.

In Australia, approximately 770 children will be diagnosed with cancer each year.^3^ It is estimated that up to 208 or 27% of these children will have a rare childhood cancer.^2^

Based on published mortality rates, it is estimated that around 25 children diagnosed with a rare cancer will die per year.^3^, ^4^ By way of comparison, in the United States approximately 10,500 children under the age of 15 will have been diagnosed with cancer in 2021.^5^ Further estimated incidences of rare childhood cancers in the United States suggest that of all childhood cancers approximately 1,600 could be classified as rare under the Foundation's eligibility criteria.^6^

Accordingly, the numbers associated with a rare childhood cancer diagnosis are very small, especially in Australia with a total population of only 25.6 million.^7^ Of the children diagnosed with a rare cancer in a 12 month period, and based upon the Foundation's definition, only 27% to 36% receive support from the Leila Rose Foundation nationally. There is a range of potential answers as to why this is the case. It could be that the Foundation's penetration of the referral base is incomplete resulting in patchy referral patterns. Another possibility is that the Foundation's definition of what constitutes a rare childhood cancer may overstate the real incidence of truly “rare,” childhood cancers. The utility of the eligibility criteria for Foundation support are constantly being evaluated and assessed.

Very early in the history of the Leila Rose Foundation the role of Family Support Coordinator was created. An experienced oncology professional, the Family Support Coordinator is available to provide support to the family for their entire cancer journey and tailor that support to their specific needs. Accordingly, the Family Support Coordinator role is unique in the pediatric cancer field in Australia in that it is confined to only rare childhood cancer, available throughout the cancer journey, with the autonomy to tailor appropriate support based upon need.

The assistance provided by the Family Support Coordinator may also include facilitating access to the Foundation's financial support. This financial assistance aims to reduce the burden of day‐to‐day expenses so that the family can focus on their child's health.

In a small number of circumstances, Leila Rose Foundation has provided additional increased funding to support new diagnostic approaches or management based upon the recommendation of the treating oncologist. This has included funding to assist a personalized medicine approach, which is being formally evaluated in Australia via the PRISM study,^8^ the results of which are eagerly awaited. The need for the Foundation to provide funding for genomic analysis has been relieved in the past few years by the Australian government's Genomics Health Futures Mission funding.^9^

The Foundation does not generally view advocacy as an important part of its role rather preferring to provide navigational support and work in conjunction with the pediatric care team to achieve the best outcomes for the patient and their family.

## 3 DATA AND OBSERVATIONS FROM ENROLLMENT RECORDS

As at 1 November 2020, 197 eligible families supporting a child diagnosed with a rare childhood cancer have been provided with assistance by the Leila Rose Foundation.

As highlighted above, support is provided for families with children less than or equal to 14 years of age at diagnosis who:

- are a citizen or permanent resident of Australia, and
- have been diagnosed with a rare childhood cancer with an incidence equal to, or less than 5% of all childhood cancers in Australia per year, ass documented in the “Childhood Cancer Incidence in Australia, 1983 to 2015” report published by the Cancer Council of Queensland.^1^, ^4^

Qualification for Foundation support is determined by a formal application to the Eligibility Sub‐Committee, which consists of the Founding Director who is a family general practitioner, a second family physician, a registered nurse and a consumer representative.

Since establishment in 2011, the Leila Rose Foundation has distributed funds of over AUD$825,000 to eligible families, largely to assist with day‐to‐day expenses so that the family can focus on their child and the management of their rare cancer.

The financial support provided by the Foundation consists of two main tiers as follows:

1. Tier 1: $2,500 financial assistance with day‐to‐day bills and incidental expenses per year for 2 years from the date of acceptance.
2. Tier 2: Up to $10,000 for a limited number of recipients to support whole genome analysis or other tailored management approaches as deemed appropriate by the treating oncologist.

Over the past decade, a total of 6 families or 3% of all supported families have received Tier 2 funding for a total of AUD$51,000 representing 6% of total funds distributed.

As highlighted above, the Medical Research Futures Fund as part of the Australian government's Genomics Health Futures Mission initiative has relieved the need for support of genomic analysis more recently.^9^

Many scientific reviews and analyses point to the impact of financial toxicity, on cancer outcomes in the United States of America and Australia.^10^, ^11^, ^12^, ^13^ While these analyses have focused mainly on adult cancer, Santacroce and Kneipp focused on the impact of financial toxicity in pediatrics and highlight the indirect impact on the affected child.^14^ The Foundation has been mindful of the financial impact of a rare childhood cancer diagnosis on the family based upon the personal experience of the founding directors. Invariably, parents are required to take time away from work to care for their sick child and this adds to pressure and stress for the family as a whole. This appears to be particularly pronounced for many families located in rural and remote communities due to the need to attend large specialized metropolitan children's hospitals.

The Foundation's enrollment records for children diagnosed with a rare cancer are shown in Table 1. Due to the small numbers, and the fact that these data subsets do not represent true population‐based frequencies, care must be taken when interpreting the data; however, there appear to be some interesting themes never‐the‐less.

**TABLE 1:** All supported patients by diagnosis and age range at acceptance

Cancer diagnosis	Number	% of total	Acceptance age range	Mean age at acceptance (y/mo)
Adrenocortical carcinoma	3	2%	1‐9 y	4 y, 8 mo
Alveolar rhabdomyosarcoma	3	2%	6‐12 y	8 y, 4 mo
Anaplastic ependymoma	1	1%	–	3
Angiosarcoma	1	1%	–	7 mo
Aplastic ependymoma	1	1%	–	3
Atypical teratoid rhabdoid tumor	6	3%	1‐4 y	2
Burkitt's leukemia	1	1%	–	5
Cardiac myxofibrosarcoma	1	1%	–	5
Cardiac sarcoma	1	1%	–	10
Choloangiocarcinom	1	1%	–	13
Chondroblastic osteosarcoma	1	1%	–	14
Choroid plexus neoplasm	2	1%	3‐6 y	4 y, 6 mo
Colorectal carcinoma	1	1%	–	15
Craniopharyngioma	1	1%	–	9
Desmoplastic small cell tumor	1	1%	–	13
Diffuse intrinsic pontine glioma	13	7%	3‐11 y	5 y, 9 mo
Diffuse midline glioma	1	1%	–	5
Disseminated glioneuronal tumor	1	1%	–	4
Embryonal pelvic rhabdomyosarcoma	1	1%	–	7 mo
Embryonal rhabdomyosarcoma	5	3%	4 mo‐5 y	1 y, 10 mo
Embryonal tumor with multilayered rosettes	4	2%	2‐4 y	2 y, 9 mo
Ependymoma	7	4%	1–9 y	5 y, 3 mo
Epithelioid sarcoma	1	1%	–	9
Ewings sarcoma	19	10%	4‐14 y	10 y, 3 mo
Gamma/delta T‐cell leukemia	1	1%	–	2
Germ cell tumor	1	1%	–	5
Glioneuronal tumor	1	1%	–	10
Hepatic angiosarcoma	1	1%	–	3
Hepatoblastoma	5	3%	1–9 y	3 y, 7 mo
Glioma	2	1%	9‐12 y	10 y, 6 mo
Histiocytic sarcoma	1	1%	–	13
Juvenile myelomonocytic leukemia	3	2%	1‐3 y	2
Langerhans cell histiocytosis	2	1%	2‐10 y	6
Malignant peripheral nerve sheath tumor	1	1%	–	16
Malignant rhabdoid tumor	2	1%	10 mo‐1 y	11 mo
Medulloblastoma	19	10%	9 mo‐12 y	6 y, 8 mo
Melanotic neuroectodermal tumor	1	1%	–	2
Metastatic alveolar rhabdomyosarcoma	2	1%	7 mo‐3 y	1 y, 9 mo
Metastatic osteosarcoma	1	1%	–	13
Muco‐epidermoid carcinoma	1	1%	–	9
Neuroblastoma	2	1%	1‐11 y	6
Orbital rhabdomyosarcoma	1	1%	–	8
Osteosarcoma	14	7%	4‐15 y	10 y, 9 mo
Pilocytic astrocytoma	1	1%	–	5
Pineal anlage tumor	1	1%	–	3
Pineoblastoma	1	1%	–	3
Primitive neuro ectodermal tumor	2	1%	1‐10 y	5 y, 6 mo
Relapsed Wilm's tumor	1	1%	–	5
Relapsed ependymoma	1	1%	–	5
Relapsed medulloblastoma	1	1%	–	4
Relapsed rhabdomyosarcoma	1	1%	–	10
Retinoblastomas	10	5%	6 mo‐4 y	1 y, 11 mo
Rhabdoid tumor	7	4%	6 mo‐2 y	1 y, 2 mo
Rhabdomyosarcoma	12	6%	5 mo‐13 y	6 y, 4 mo
Steroid cell tumor	1	1%	–	8
Synovial sarcoma	1	1%	–	15
Thalmic glioma	1	1%	–	5
Wilms tumor	18	9%	2‐12 y	4 y, 3 mo
Total	197	100%		6 y

In almost 10 years of enrollment, a total of 197 patients diagnosed with a rare childhood cancer have been accepted for support with an average age of 6 years at acceptance.

The most frequent rare childhood cancer diagnoses have included:

- Medulloblastoma—20 patients (including an additional relapsed patient representing 10% of total enrollments).
- Ewing's sarcoma—19 patients (10% of total enrollments).
- Wilm's tumor—19 patients (including an additional relapsed patient representing 10% of total enrollments)
- Osteosarcoma—14 patients (7% of total enrollments)
- Diffuse intrinsic pontine glioma—13 patients (7% of total enrollments)

Overall, 35 patients representing 18% of all enrollments have had a unique diagnosis that has not been recorded for any other enrolled patient, highlighting that these diagnoses are in themselves, extremely rare.

While no conclusions can be drawn with respect to age ranges by acceptance into the Foundation, these data are generally in line with international published data for age at diagnosis. For example, the Foundation's enrollment data show that rhabdoid tumors presented with a range at enrollment of 6 months to 2 years and a mean average of 1 year, 2 months, which is consistent with international data.^15^ Similarly, the Foundation's figures illustrate that atypical teratoid rhabdoid tumor patients have been enrolled with an age range of 1 to 4 years and a mean average of 2 years which is again in line with the published literature which reflects a peak diagnosis at less than 3 years of age.^16^ Retinoblastomas, have an age range of 6 months to 4 years at enrollment with a mean average of 1 year, 11 months which is consistent with the data provided in the American Cancer Society, Cancer Facts & Figures 2020 suggesting that the average age of children at diagnosis is 2 years.^17^ If nothing else this supports the idea that for these tumors at least, the period between diagnosis and enrolment with the Foundation is very short.

The remaining tumor types are generally associated with broader age ranges or the numbers are too small to draw out any themes.

**FIGURE 1:**
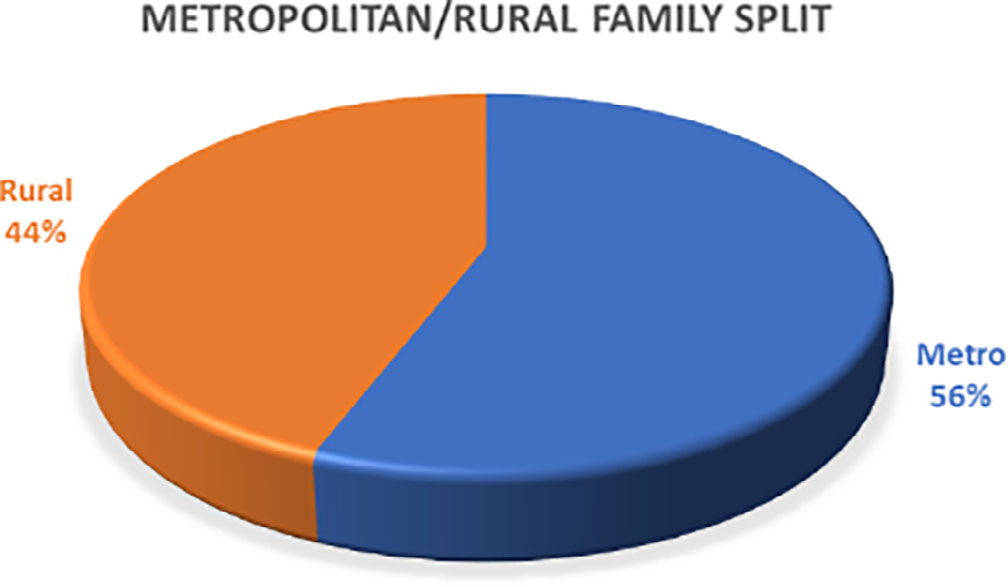
Metropolitan vs rural and regional split for all patients (N = 197)

The Foundation provides support for families in metropolitan as well as regional and rural areas. For the purpose of our analysis postcodes (Zip codes) outside of the major metropolitan boundaries across Australia are defined as “rural.” As highlighted in Figure 1, the metropolitan: rural split for the Foundation‐supported patients is 56% and 44% respectively. Rural and regional families have particular challenges as they are often located a long distance from the major city‐based treatment centers and family members may be required to relocate or travel over long distances for treatment, assessment or ongoing management adding to the stress on the family unit as a whole.

It is estimated that 29% of the Australian population live in rural and remote areas.^18^ Accordingly, the proportion of families provided with support by the Foundation is significantly above the population potential underlining the greater apparent level of need among rural and remote families. This observation requires further investigation to fully understand the needs and motivations at play.

Notwithstanding the above observations, there is no restriction on enrolment with the Foundation from an ethnic, socioeconomic or location‐perspective. Enrollment is largely determined by the referring cancer center as long as the diagnostic and age‐range criteria are met.

Table 2 shows the metropolitan‐rural split by age. Both groups are closely matched with respect to age suggesting that there is not a great deal of difference between detection rates of rare cancers between the city and rural areas in Australia.

**TABLE 2:** Metropolitan vs rural split by age

	Number	Average age (years, months)
Metro	110	6 y 1 mo
Rural	87	5 y 11 mo
Total	197	6 y

## 4 THE ROLE OF THE FAMILY SUPPORT COORDINATOR

Facilitated by direct contact with individual families, the Family Support Coordinator is uniquely placed to determine and tailor ongoing personalized support based upon each family's specific needs and circumstances.

An important element of the tailored support provided by the Foundation's Family Support Coordinator includes “navigational,” support. “Navigation,” involves presenting the options and range of health services that may be accessed by a family throughout the twists and turns of their child's cancer diagnosis and ongoing transitions of care.

In order to deliver streamlined, family centered care, the Family Support Coordinator works both independently and in collaboration with physicians and multidisciplinary teams at the child's treating hospital to achieve optimal outcomes. To ensure that the Foundation's families have a complete and sound understanding of their child diagnosis and treatment options, the Family Support Coordinator where appropriate, accompanies families to appointments with the medical oncologist as an objective facilitator and a medical jargon interpreter. This assists in clarifying, and explaining information from the oncology care team, so that the family is able to make informed decisions about their child's care. Each family has different needs and the skill of the Family Support Coordinator lies in their ability to tailor solutions according to those needs. The following case studies provide some real‐life examples.

### 4.1 Case study‐a personalized medicine approach

In 2016, Patient 1 (P1) was a 6 year‐old healthy boy, growing up in rural New South Wales, Australia. He rarely complained about pain, so when he came home from school distressed, limping, and complaining of severe pain in his left leg his parents took it seriously. On observation, his parents noticed that his left foot was discolored, pale and cool to the touch. P1 was immediately rushed to the local emergency room. Doctors quickly identified that P1 had a heart murmur and a suspected aortic valve infection, which had thrown off a clot into his femoral artery causing his pain.

P1 was urgently flown by helicopter to Westmead Children's hospital in Western Sydney, 250 km away, where he underwent an urgent echocardiogram. The echocardiogram showed a large mass in the aorta, a blood clot and what appeared to be a mass in his aortic artery. P1 was prepared for surgery and that night the cardiac team went on to perform a Ross procedure whereby they used his pulmonary valve to replace his extensively damaged aortic valve. They then used a biological tissue valve to replace his damaged pulmonary valve. Due to his heavily compensated coronary artery, surgeons discovered P1 had developed a compensatory circulation that perfused his heart effectively and kept him alive. The vascular team then also performed an embolectomy on his upper thigh and a fasciotomy on his lower left leg. Post‐surgery, P1's family was told that his aortic valve appeared to be compromized by a tumor and not by an infection as first thought. The initial pathology report came back as inconclusive and with more rigorous investigations P1 was finally formally diagnosed with Malignant Myxofibrosarcoma.

According to P1's specialists, malignant myxofibrosarcoma is almost exclusively seen in the extremities of the elderly. This was an unprecedented case. The surgical team was unable to identify clear margins, and accordingly could not perform further surgery.

P1's specialists explained that this type of cancer was not responsive to chemotherapy and that the dose of radiation required to successfully treat his disease would significantly damage his heart. Contrasting management alternatives of watch and wait against VAC chemotherapy were considered but subsequently dismissed by P1's parents in consultation with his medical team.

As a last resort, the family agreed to P1's tumor being sent off for genomic analysis in the United States in the hope of finding a genetic target for treatment. The oncologist stated to the family that the odds of finding anything valuable from this approach were long and the analysis expensive. Without hesitation the family agreed to the genomic analysis and tumor profiling. The next struggle for the family was raising the AUD$7,000 required for the analysis at such short notice, particularly as they were already experiencing significant financial difficulty due to P1's diagnosis. Importantly, this case arose approximately 1 year prior to the commencement of the PRISM trial in Australia, which would have employed genomic analysis as part of the protocol.

P1's oncologist knew that Leila Rose Foundation offered financial support to families with children with rare cancer, so referred them onto the Foundation through the Family Support Coordinator. P1's application was expedited to the Foundation's board for consideration for tier 2 funding of up to AUD$10,000. The board quickly approved the family's request for financial support.

The genomic analysis identified a genetic target and with this information, a tailored treatment consisting of oral Sorafenib tosylate was prescribed.

Four years later, P1 is disease‐free, off Sorafenib and living the full life of any other 10‐year‐old boy. For everyone associated with this case, it was a wonderful outcome and a great team effort.

### 4.2 Case study‐assistance provided post‐treatment when care needs were greatest

Patient 2 (P2) is a three‐year‐old boy, who lives with his parents, and younger brother in metropolitan Melbourne. P2's mother began to notice that something was wrong with P2's vision, when they would play hide and seek. He would ask where his mother was when she was standing directly in front of him. He would also continuously run into walls and objects that were in plain sight, and when reading he was not able to discern pictures of his favorite characters in picture books. In a dimly lit room, his mother also noticed a white glow from his pupil. Concerned, P2's mother, took him to the local doctor where they suggested that he may be color blind or that he required glasses, so P2 was referred to an ophthalmologist. Following the doctor's appointment, that night P2 complained of a severe headache and so P2's mother took him to the Royal Children's Hospital, emergency room, where he saw a specialist pediatric ophthalmologist. The ophthalmologist requested a number of assessments and with those, P2 was confirmed as having severe bilateral retinoblastoma.

With the confirmation of his rare cancer diagnosis, P2's mother self‐referred him to the Leila Rose Foundation via the Family Support Coordinator. His application was accepted.

P2 completed six cycles of high‐dose chemotherapy to control his disease and preserve as much vision as possible. The chemotherapy was augmented by laser therapy and cryotherapy delivered directly into the tumors in his eyes. Concurrent monthly examinations of his eyes were conducted under general anesthetic. In July 2020, P2's left eye had to be enucleated in order to limit the spread of the tumor. The surgery was successful. Despite aggressive management, P2 still had retinoblastoma in his right eye resulting in poor vision and had to adjust to life with total loss of eyesight on his left‐hand side. P2's mother contacted the Family Support Coordinator requesting assistance in locating vision impairment services.

The Family Support Coordinator provided P2's mother with information on the “Royal Institute for Deaf and Blind Children.” Located close to the family's home the institute provided parental education, one to one intervention therapy, allied health and vision clinical services suitable for P2's individual needs.

The Family Support Coordinator also put P2's mother in contact with some occupational therapy outlets that provided “hands‐on,” games and activities while the financial support offered by the Foundation helped the family to cover the cost of an indoor playset for P2, as well as the games and activities. These items not only helped to assist P2 in developing his fine motor skills but also taught him about colors while he still had vision. They were also delivered to P2's family in the midst of the 2020 Covid‐19 pandemic, which meant that P2 and his younger brother had safe, indoor activities away from potential exposure to Covid‐19. This was particularly important as P2 was immunocompromized as a result of his chemotherapy. His parents were extremely grateful for this support. His mother commented:

“I'm so thankful to the Leila Rose Foundation for sending Gemma (the Family Support Coordinator) into our lives. Her advice, support, and just remembering the little details I once mentioned makes me feel like I matter. Knowing that I can always come to her has been a much‐needed breath of fresh air!”

### 4.3 Case study‐support in the face of financial toxicity

Since the establishment of Leila Rose Foundation in 2011, it has become apparent that “financial toxicity” can have a profound negative impact on families affected by rare childhood cancer. This is why financial support with day‐to‐day expenses has been a focus of the Foundation's support from establishment.

In 2013, Patient 3 (P3) was a very petite, healthy young 6‐year‐old girl, however when she became unwell, she was rushed to the local regional hospital where she was found to have a mass on her brain. Transferred to Royal Children's Hospital Melbourne, 300 kms away, P3 underwent tumor debulking surgery, with approximately 80% of her tumor being removed. Despite successful surgery, her specialist was unable to determine a definitive diagnosis, due to inconclusive biopsy results. To assist in P3's diagnosis, case notes including tissue samples were sent to pathology specialists in the United States. They too were unable to provide a definitive diagnosis. Eleven days after P3's de‐bulking surgery an MRI of her head and spine revealed that she had further disease.

After a lengthy treatment discussion, P3's family opted to commence 4 weeks of vincristine‐carboplatin chemotherapy. With further MRI and methylation analysis, P3's specialists formally diagnosed her with Metastatic Pilocytic Astrocytoma (a cancer no longer funded by the Foundation as it now accounts for 9.5% of total pediatric cancer diagnosed per year). Despite P3 experiencing some initial disease progression, the chemotherapy appeared to halt the growth of all visible tumors, with two tumors reducing in size. Never the less, P3 required secondary tumor debulking surgery, which resulted in her hypothalamus being severely damaged and her tumor bleeding into her brain. The result was that she now had an acquired brain injury that resulted in her experiencing extreme hunger 24 hours a day. In the ensuing 6 weeks P3 gained a significant amount of weight.

As a result of the secondary surgery P3 had developed Diabetes Insipidus which saw her being hospitalized for a total of 189 days over a twelve‐month period. Patient management and assessment became increasingly difficult. She developed extreme hypersensitivity to all stimuli. She was unable to walk barefooted without discomfort. She also became very distressed by loud noises and experienced severe pain in her shoulders. After lengthy discussion with P3's specialist, her family opted not to radiate her whole brain due to the serious side effects and potential cerebral injury. She was referred for palliative therapy.

Throughout P3s cancer journey, the family were faced with numerous financial stressors. The need to take a significant amount of time off work to care for her saw the family suffer significant financial hardship. Being far away from P3s main treating hospital alone resulted in substantive travel costs. The family was unable to pay for their car registration in addition to utility and phone bills. Leila Rose Foundation was able to assist the family in paying for fuel vouchers as well as covering their utility bills and incidental expenses.

The family also needed to buy aids such as orthotic shoes for P3's sensitive feet as well as a new posture‐supporting bed that would be suitable for her increased weight and obstructive sleep apnea. The Family Support Coordinator was able to help the family source these items with the Foundation paying for them, so that P3's family could stay afloat financially.

P3's parents commented:

“We are very touched by the generosity of the Leila Rose Foundation and the invaluable support you have shown our family through the years. You have made a big difference to our lives and we feel blessed. Thank you!

### 4.4 Case study‐a second opinion

In a small number of cases the support provided by Leila Rose Foundation may include referring families to the “Best Doctors” network. This process is undertaken with the acknowledgement and in collaboration with the child's treating specialist. The Best Doctors network is drawn upon when very little is known about the child's cancer or when treatment options are very limited. This approach is in line with the Foundation's philosophy of “leaving no stone unturned.” The “Best Doctors” organization draws on a panel of medical opinion leaders from around the world that specialize in very specific health issues, in this case rare childhood cancer.

In early 2015, Patient 4 (P4) had just turned 14 and was thriving. Her mother became concerned when she started to complain of back pain and took her to a local health center where routine x‐rays discovered she suffered from scoliosis. P4 was then referred to an orthopedic specialist who provided her with a back brace. Eight months later at a routine check‐up, further X‐rays revealed that P4 had suspicious lesions throughout her spine, two on her ribs and one on her right lower brain stem. Further imaging and a biopsy at the local children's hospital led to a diagnosis of multifocal histiocytic sarcoma. Due to the rarity of the cancer and there being little evidence of successful treatment plans, P4's family, in collaboration with their specialist sought advice from a leading international medical expert. On the basis of this advice, P4 commenced an aggressive treatment regimen consisting of chemotherapy, radiotherapy and thalidomide. It was early 2016, and P4 and her family were referred by their social worker to the Leila Rose Foundation, for support.

Despite the aggressive regimen, her tumors failed to respond. As her mother's concerns grew, and P4's health deteriorated, P4's mother contacted the Family Support Coordinator and requested to be referred to “Best Doctors,” to seek a second medical opinion. Her mother commented at the time,

“When P4 was diagnosed, I was full of questions and concerned. I needed someone to have a second look at the case, in the hope that other treatment would be available.”

Best Doctors contacted P4's treating specialists seeking access to her scans and medical history. The Best Doctors team also requested further tissue pathology to be sure of the diagnosis. Their findings were different from the original diagnosis of multifocal histiocytic sarcoma. To be sure the conclusions were crossed checked with a second “Best Doctors” oncology panel who agreed with the new diagnosis of Pseudomyogenic Hemangioendothelioma, an even rarer soft tissue tumor.

From these findings, Best Doctors recommended a differing treatment plan, which involved radiotherapy and surgery alone. The conclusion drawn by Best Doctors was presented to P4's local specialist who then too, formally changed her diagnosis to Pseudomyogenic Heamangioendothelioma and the new treatment was commenced.

Sadly, despite the best efforts of the family and P4's care team, she lost her battle with her cancer and passed away in late 2016. The second opinion had spared P4 from the ongoing toxicity of further chemotherapy, during her final months. By utilizing Best Doctors, P4 and her mother had peace of mind knowing that “no stone was left unturned” in P4's cancer battle.

## 5 AN ONGOING FOCUS TO LEAVE NO STONE UNTURNED

The above cases, exemplify the diverse needs of a small number of families affected by rare childhood cancers. In Australia, Leila Rose Foundation and other cancer charities and associated services, support one another and collaborate where possible to achieve optimal patient outcomes from diagnosis to bereavement or survivorship. Although the Foundation's scope of practice is broad, there may at times be a ready‐made solution for a particular family's situation. It is the Family Support coordinator's role to source and recommend appropriate support services to ensure the best possible health and wellbeing outcomes.

Additional tier 2 funding has also been utilized in a small number of cases, whereby Leila Rose Foundation via the Family Support Coordinator, works in collaboration with the family's oncologist providing financial support for chosen management approaches, which may not be reimbursed under the Australian health system.

Unfortunately, despite the treating team and the Foundation's best efforts, approximately 25 Australian families with a child diagnosed with a rare cancer will be told that nothing can be done for their child in a given year.^4^ One of the key roles of the Family Support Coordinator is to provide informal emotional and bereavement support, by providing an empathetic, listening ear or where appropriate referring onto psychological services and peer support groups.

## 6 CONCLUSION

The diagnosis of a rare childhood cancer is a shattering experience for a family. The Leila Rose Foundation, through the Family Support Coordinator seeks to ensure that no stone is left unturned while reducing the financial pressures of day‐to‐day expenses that distract from the battle against the cancer that threatens their child's precious life. Rare childhood cancer by its very nature remains a difficult area for research, where the extremely small number of patients globally limits the scope for controlled studies. The outcome for many children diagnosed with childhood cancer has improved substantially over the past decade, however due to the small numbers and the lack of specific epidemiological data no conclusions can be drawn in terms of survival of children affected by rare cancers.^19^ Notwithstanding this, overall survival rates are still low relative to more common cancers and the Foundation is committed to delivering its unique support services to families now and in the future with a view to reducing the burden on families and particularly with respect to the financial toxicity which often accompanies a rare childhood cancer diagnosis.

## AUTHOR CONTRIBUTIONS

**G.S.:** Data curation; writing‐original draft. **A.C.:** Resources; supervision; validation; visualization. **T.C.:** Data curation; validation. **C.B.:** Conceptualization; data curation; formal analysis; project administration; writing‐original draft; writing‐review & editing.

## CONFLICT OF INTEREST

No proceeds from donations, grants or other financial support were used to fund this publication. Christopher Broadley is employed part‐time by ITL Australia Pty Ltd—An Australian‐based medical devices organization in addition to his role as CEO of the Leila Rose Foundation. There is no conflict of interest for these roles, which are approved by the boards of both organizations. None of the other authors has potential conflicts of interest to declare.

## ETHICS STATEMENT

Patient confidentiality has been maintained in all data tables and case studies contained in this brief report. Never‐the‐less, families whose children have been included in the case studies have been consulted where possible and the details verified. Leila Rose Foundation is a nationally registered charity and not‐for‐profit organization. The Foundation does not receive government funding or assistance and is financed primarily by community grant, personal donations, and fund raising activities.

## Data Availability

Data was tabulated from historical applications for support using the Foundation's approved application process. All data was de‐identified and provided in a summarized format for this brief report.
